# Supplementary figures and images for: Red deer synchronise their activity with close neighbours
Source: PeerJ. 2014 Apr 10;2:e344. doi: 10.7717/peerj.344 (PMC3994637; doi:10.7717/peerj.344)

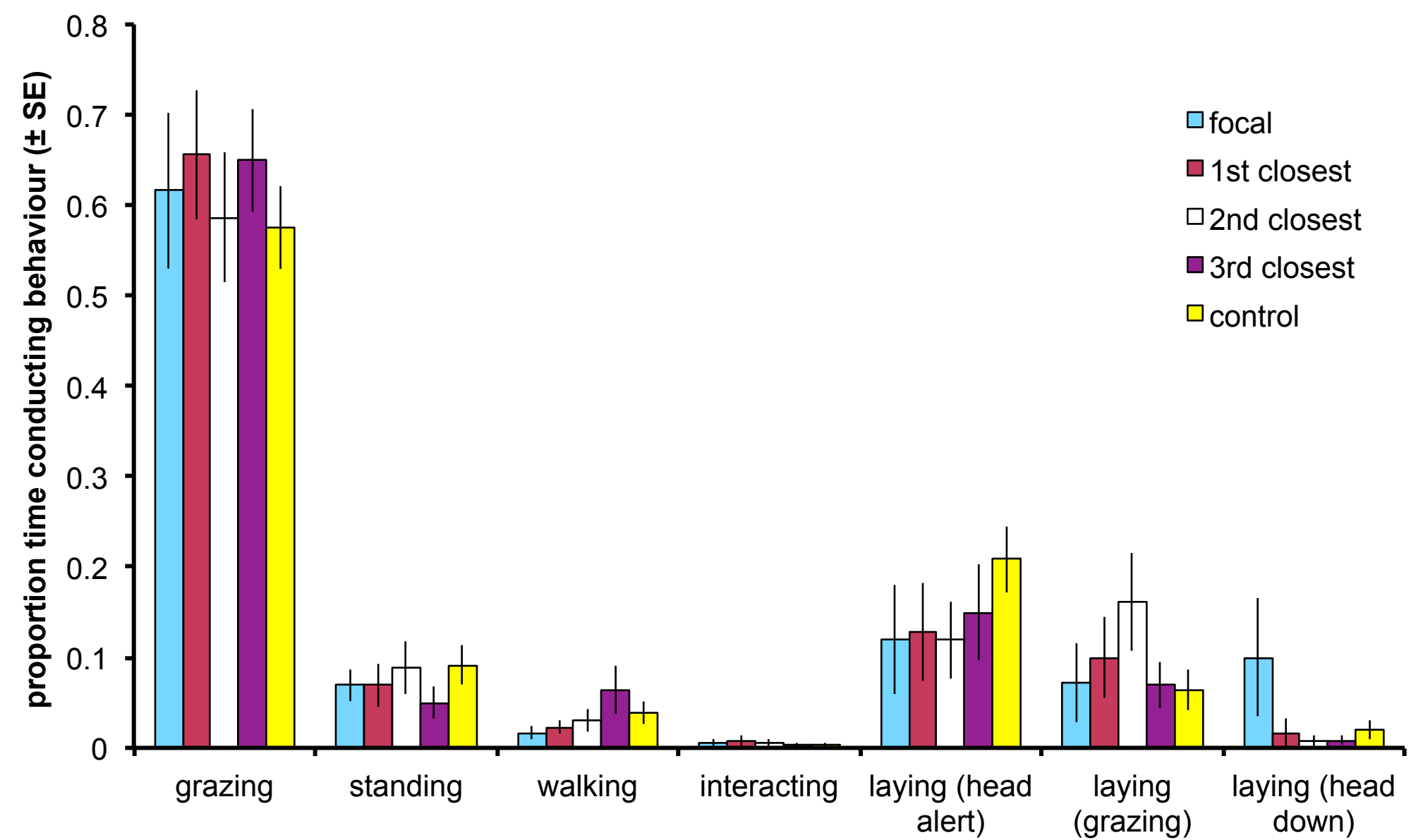

Supplement: Supplemental Information 2 [file peerj-02-344-s002.pdf]

**proportion of time  
conducting identical behaviour**

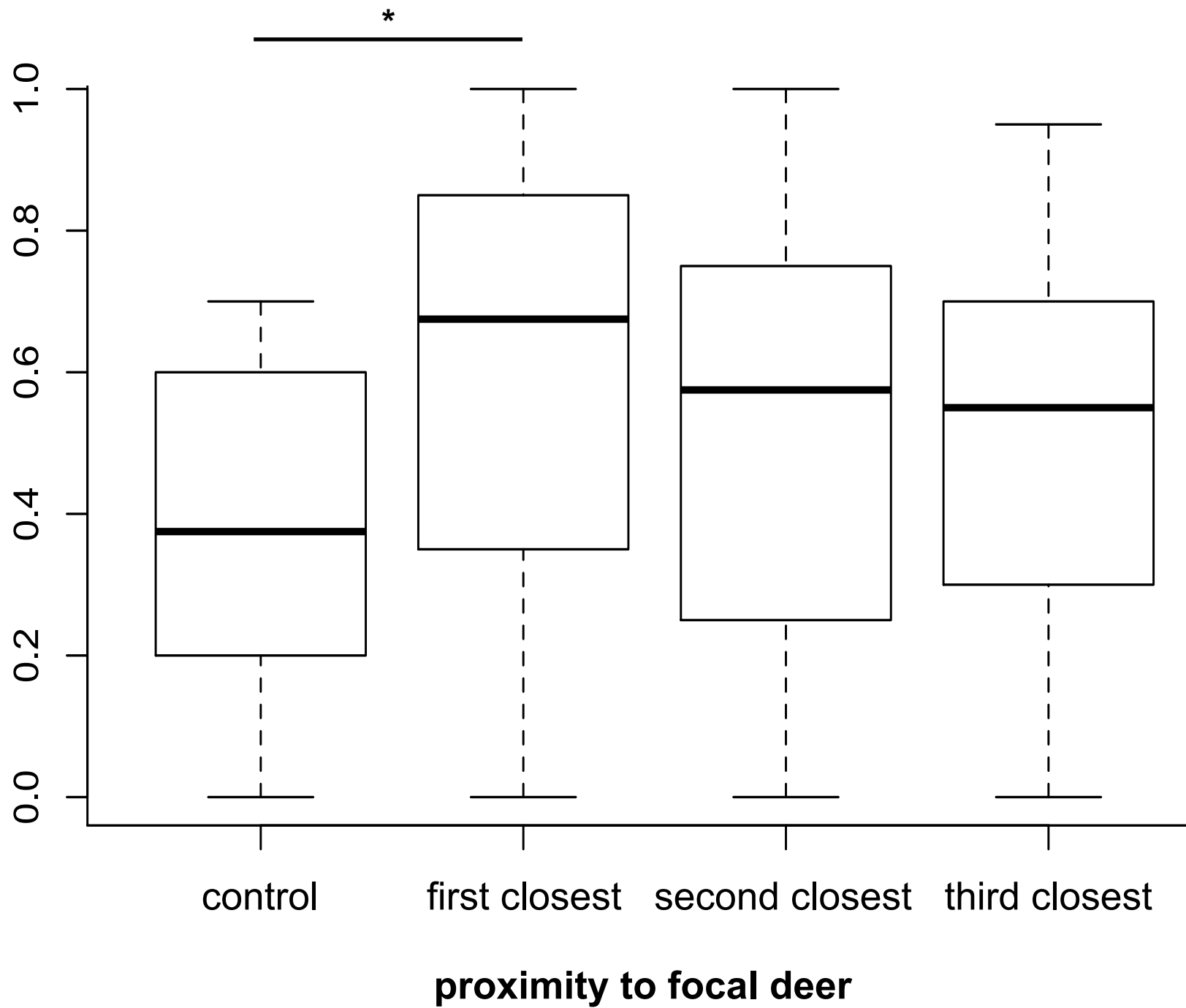

Supplement: Supplemental Information 3 — Synchronisation of individual behaviours between the focal individual and its neighbours or a control individual was assessed by calculating the proportion of observation periods that each focal individual was conducting exactly the same behaviour as the compared individual, considering each of the eight possible behavioural classes recorded as a different behaviour. Data were compared using a Friedman test, as they did not fit assumptions of normality, and post hoc analyses were conducted using two-tailed Wilcoxon signed-ranks tests assuming a normal approximation with continuity corrections, with the significance value adjusted to p = 0.009 using a Bonferroni correction. Deer at different social distances differed in their level of synchronisation with the focal individual (${\chi }_{3}^{2}=19.29$χ32=19.29, p < 0.001), and pairwise comparisons demonstrated that the focal individuals were more likely to be synchronised with their closest neighbour than with a control individual (p = 0.001); all other pairwise comparisons were non-significant. [file peerj-02-344-s003.pdf]
